# A Blockchain-Enabled Framework for mHealth Systems

**DOI:** 10.3390/s21082828

**Published:** 2021-04-16

**Authors:** Dragos Daniel Taralunga, Bogdan Cristian Florea

**Affiliations:** 1 Faculty of Electronics, Telecommunications and Information Technology, Politehnica University of Bucharest, 060042 Bucharest, Romania; bogdan.florea@upb.ro; 2 Faculty of Medical Engineering, Politehnica University of Bucharest, 060042 Bucharest, Romania

**Keywords:** mHealth, blockchain, wearable sensors, IoT, smart contract, Ethereum, IPFS

## Abstract

Presently modern technology makes a significant contribution to the transition from traditional healthcare to smart healthcare systems. Mobile health (mHealth) uses advances in wearable sensors, telecommunications and the Internet of Things (IoT) to propose a new healthcare concept centered on the patient. Patients’ real-time remote continuous health monitoring, remote diagnosis, treatment, and therapy is possible in an mHealth system. However, major limitations include the transparency, security, and privacy of health data. One possible solution to this is the use of blockchain technologies, which have found numerous applications in the healthcare domain mainly due to theirs features such as decentralization (no central authority is needed), immutability, traceability, and transparency. We propose an mHealth system that uses a private blockchain based on the Ethereum platform, where wearable sensors can communicate with a smart device (a smartphone or smart tablet) that uses a peer-to-peer hypermedia protocol, the InterPlanetary File System (IPFS), for the distributed storage of health-related data. Smart contracts are used to create data queries, to access patient data by healthcare providers, to record diagnostic, treatment, and therapy, and to send alerts to patients and medical professionals.

## 1. Introduction

Mobile communication, mobile devices, and the Internet of Things (IoT) have changed entire sectors, such as education, transportation, agriculture, etc. The use of IoT, through which people, processes, data, and devices connect to each other over the Internet, is experiencing considerable growth: mobile machine-to-machine (M2M) connections are expected to grow from 1.2 billion in 2018 to 4.4 billion by 2023 [1]. Also, the number of smartphones is forecast to grow from 4.9 billion in 2018 to 6.7 billion by 2023 [1].

Presently, mobile technology is reshaping the traditional healthcare model into the so-called mobile health (mHealth) model, placing the patient at the center of the healthcare system and motivating patients to assume responsibility for their own wellbeing [2]. A complete mHealth system consists of interconnected wearable sensors, IoT services, mobile devices, mobile applications, and cloud services. The interconnected wearable sensors form a Wireless Body Area Network (WBAN) and facilitates acquisitions of biomedical signals (e.g., electrocardiogram—ECG; phonocardiogram—PCG; photoplethysmogram—PPG) and biomedical parameters (e.g., heart-rate, blood pressure, respiration rate, blood oxygen saturation, energy expenditure, etc.) which are transmitted wirelessly to mobile devices. Thus, mHealth systems enable remote patient monitoring, rehabilitation, therapy, diagnosis, and treatment. The huge amount of data generated in mHealth systems can be used to perform complex analysis of patients’ physical, cognitive, and physiological conditions, thus facilitating predictive and preventative healthcare. Moreover, the health data collected from groups of patients through mHealth systems can be used in medical research (clinical trials, randomized control trials, etc.).

In mHeatlh systems, because sensitive data is recorded, analyzed, shared, and stored, the main challenges are data provenance, access control, data integrity, and identity management. However, a major limitation of mHealth systems is public trust, mainly due to possible security vulnerabilities that would allow medical data alteration, unauthorized sharing, data theft, data loss, etc.

In the present paper, a blockchain-based mHealth framework is proposed that addresses security, data integrity, and data provenance challenges. Blockchain technology has important features, such as traceability, transparency, decentralization (no central authority is needed), and immutability. At the time of writing, there is no dedicated framework described in the literature based on blockchain for a complete mHealth system that also allows medical experts to directly interact with medical signals collected by wearable sensors to determine a diagnostic. As discussed in the next sections, medical applications where blockchain technology is most used presently are electronic health records (EHR). The core contributions of the proposed solution are:

- The design, implementation, and deployment of the blockchain network and the smart contract;
- Data modeling and integration with the InterPlanetary File System (IPFS);
- The implementation of a bidirectional functionality that offers the possibility for medical experts and patients to upload and monitor data in a continuous manner;
- The implementation of an interface that permits medical experts to extract diagnostic information by interacting with physiological signals available on the proposed mHealth system.

The paper is structured as follows: in Section 2, the concept of mHealth is introduced, emphasizing the role of wearable sensors and the challenges of mHealth systems; in Section 3, the blockchain technology is described; the proposed mHealth framework is detailed in Section 4, and the results, discussion, and conclusions are described and presented in Section 5, Section 6 and Section 7.

## 2. Evaluation of Mhealth Systems

Presently, traditional models for delivering healthcare to patients are shifting into the digital health era, mainly thanks to advances in information and communication technologies such as Big Data (BD), Artificial Intelligence (AI), Deep Learning (DL), Machine Learning (ML), 5G technology, the Internet of Things (IoT), etc. mHealth systems are part of new digital health, and are transforming and revolutionizing the prevention, diagnosis, treatment, recovery, or cure of disease, illness or injury, the interaction between medical staff and patients, medical models (changing from disease-centered to patient-centered care [3]), and medical management (changing from general to personalized management [3]), etc. Through Internet-connected mHealth devices and sensors, medical staff have omnipresent access to health data (enabling earlier disease detection and prevention), while patients can access and share health information and receive health counseling. By offering the possibility of home monitoring, mHealth has also had a significant impact on the expense of healthcare.

**Figure 1:**
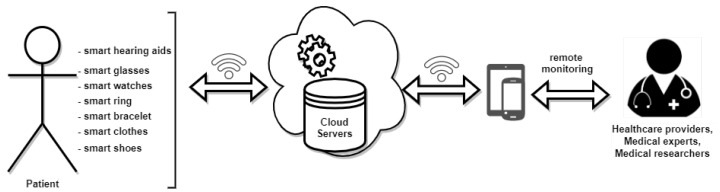
General architecture of an mHealth system.

As described in Figure 1, a complete mHealth service system comprises different technologies:

- **Wearable devices and sensor technology** are used to monitor different biomedical parameters and signals (e.g., heart-rate, respiration rate, blood pressure, oxygen saturation levels, eye movement, gait, foot pressure distribution and bio-potentials such as electrocardiogram (ECG), electromyogram (EMG), electroencephalogram (EEG), etc.);
- **IoT**: Wearable devices are connected, usually in a wireless network, and can exchange information between each other and with other devices and systems over the Internet;
- **Cloud servers and services**: the volume of data generated by an mHealth system is usually stored in the cloud. Using BD, DL, or ML, the data can be analyzed, and deep insights about the health of the patient can be derived. Data can be also archived and used to train DL or ML models to predict the development of different diseases and to recommend possible care, treatment, and therapy [4];
- **Mobile devices and applications**: devices such as smart mobile phones and mobile tablets, together with specially designed health mobile applications can be used to visualize and interact with the patient data, to diagnose and offer health counseling and treatment.

mHealth systems can be used for a very wide range of healthcare applications, and some of the most common categories are discussed below.

- **Cardiopulmonary monitoring**: signals such as phonocardiogram (PCG), electrocardiogram (ECG) [5], and photoplethysmogram (PPG) [6] are recorded, and vital biomedical parameters are derived, including heart-rate (HR), heart-rate variability (HRV), health of heart valves, blood pressure, respiration rate, blood oxygen saturation, chest volume variation, etc. Analysis of these bio-potential signals and biomedical parameters allows earlier detection and prevention of cardiovascular diseases, and helps both medical experts and patients manage cardiovascular problems better. mHealth systems are used to help manage cardiopulmonary diseases such as hypertension, arrhythmia, coronary artery disease and heart failure, myocardial infarction, pulmonary hypertension, and chronic obstructive pulmonary disease [6,7,8,9,10,11,12]. In particular, the early detection of atrial fibrillation arrhythmia, most commonly, using mHealth systems can prevent strokes and reduce hospitalizations [13,14,15]. In a recent study, a Deep Neural Network (DNN) was developed to classify 12 heart-rhythm classes using single-lead ECG signals recorded with an mHealth device. More than 50,000 patients were included in the study and the DNN was trained on more than 90,000 single-lead ECG signals. The results proved that the proposed DNN was able to classify different types of arrhythmia from single-lead ECG signals with high diagnostic performance similar to that of cardiologists [16];
- **Fitness level tracking**: regular physical exercise is the main way to prevent obesity and to maintain a healthy lifestyle. However, due to lack of time and motivation, high costs of monthly gym memberships and personal trainers, and sometimes due to special circulation restrictions (e.g., lockdown during the COVID-19 pandemic), many people choose to work out from home. In this case, mHealth systems can be used to monitor and assess workout exercises. Based on the fitness level of the user and on information collected by the sensors, the mHealth system can offer a personalized workout plan, suggestions for correct and efficient physical exercises execution, statistical feedback to help keep track of the fitness plan, etc. [17]. For such mHealth applications, usually specific parameters and information are recorded, such as heart-rate, energy expenditure, temperature, skin perspiration, plantar pressure, speed and acceleration, position of the body or a specific part of the body (e.g., hand, limb), number of repetitions, type of exercise, etc. In addition, the data collected can be analyzed to predict possible injury [18].
- **Cancer**: mHealth approaches can be used to improve screening rates for different types of cancer. In a recent review, 12 studies were analyzed, in which women were informed and reminded about their upcoming screening appointments for cervical cancer, via text message and mobile application [19]. The results showed that mHealth approaches may be an effective strategy to contact women for improving cervical cancer screening rates. The main concern of the participants was related to privacy and confidentiality aspects during the exchange of health information. In [20], the authors proposed a new mHealth system for the early detection of oral cancer in a rural population where no medical experts were available. Frontline health workers (FHP) were trained to take images with smartphones and send them to medical experts, who in turn analyzed the images and suggested a diagnostic or possible treatment. The approach was evaluated and validated on more than 45,000 subjects, during a period of eight years (2010–2018) [20,21]. Brown-Johnson et al. proposed a mHealth perspective for patients with lung cancer to manage experiences of stigma. A health game is developed that allows lung cancer patients to improve communication with their clinicians, to decrease lung cancer stigma and to obtain optimal self-management [22]. In [23], a mHealth platform was designed and implemented for tumor treatment. The wearable platform was controlled by a smartphone and targeted tumor therapy was conducted. A significant prevention of tumor recurrence and tumor growth inhibition was reported.
- **Psychiatry**: patients suffering from mental disorders such as bipolar disorder, schizoaffective disorder, and schizophrenia have a high degree of cognitive and functional impairment, which drastically reduces the quality of life. The main objectives of mHealth approaches are to improve engagement with treatment and services. Thus, with the help of mHealth technologies, the following benefits can be obtained: (i) identifying patients who are at risk; (ii) encouraging exercise and behavior change; (iii) reminding the patient of the next appointment or to take the medication; (iv) self-management techniques; (v) monitoring symptoms in real time; (vi) developing personalized interventions and caring plans; (vii) identifying warning signs based on self-reports of wellbeing; and (viii) offering continuous professional counseling [24,25]. However, there are some barriers in the use of mHealth services that patients with severe mental illness may experience, including low income, unstable housing that can influence access to the Internet, cognitive impairment, and symptoms such as apathy, depression, low motivation, and paranoia. Nevertheless, recent studies have shown that people with psychosis, despite experiencing these barriers, can make use of smart mobile technologies almost the same as the general population [24,26].
- **Rehabilitation and therapy**: there is a significant number of mHealth approaches for rehabilitation and therapy described in the literature, which can be categorized as the following, [27]: *stroke rehabilitation* (monitoring and feedback for, physical activity, cognitive assessment, education to raise awareness, education on home exercises for managing poststroke, assistive devices, cognitive assessment) [27,28,29,30]; *traumatic brain injury rehabilitation*: improving cognitive memory using gamification, using Global Positioning Systems (GPS) to make the use of transportation system easier, providing a home-based rehabilitation plan that includes training with daily activities, planning of appointments using reminders [31,32]; *pulmonary rehabilitation* (enabling home-based rehabilitation for patients with obstructive pulmonary disease (OPD), monitoring daily physical activity, offering motivational messages and/or feedback (e.g., acoustic), analyzing daily symptoms and physiological variables, detecting chronic OPD exacerbations and evaluating the clinical risk of chronic OPD) [33,34,35]; *cardiac rehabilitation* (monitoring symptoms and physical activity (via ECG recorded signals, HR, HRV, energy expenditure, etc.), improving exercise and medication, messaging with healthcare providers) [36,37,38]; *Musculoskeletal rehabilitation*: enabling wrist and/or hand rehabilitation using a sensor glove and gamification, improving motion with haptic feedback, prediction of rheumatoid arthritis activity by analyzing data related to joint symptoms, walking ability, limitations of daily activity, determining the osteoarthritis index) [39,40,41,42].

### 2.1. Role of Wearable Sensors in mHealth Systems

Wearable sensors are a key component of a mHealth systems, and are used to obtain reliable data regarding patient health, behavior, vital signs, activity, etc. They are becoming popular in many health-related disciplines. The advances in sensor manufacturing technology have allowed the development of miniature sensors that are generally non-invasive, and which are revolutionizing the entire global health system. A variety of wearable sensors exist, depending on the target information to be recorded, processed, and analyzed. When more sensors are used in an mHealth system, usually these are interconnected via some medium and they form a network of wearable sensors named an Wireless Body Area Network (WBAN) [43,43]. The sensors can exchange information between each other, and send it to a cloud server (Figure 1). Technologies that are used to interconnect wearable sensors are *ZigBee* (IEEE 802.15.4 standard; data rates—250–300 Kbps; range—100 m; network topology—mesh, star, tree, and cluster; bandwidth—2.4 GHz); *Bluetooth* (IEEE 802.15.1 standard; data rates—1–3 Mbps; range—10 m; network topology—star; bandwidth—2.4 GHz); *Wi-Fi* (IEEE 802.11 standard; data rates—1–40 Mbps; range—up to 5 km; network topology—tree, star, and P2P; bandwidth—2.4, 3.7, and 5 GHz); *WiMax* (IEEE 802.11 standard; data rates—75 Mbps; range—15 km; network topology—tree, star, and P2P; bandwidth—2.3, 2.5, and 3.4 GHz) [44]. The use of a particular wireless protocol for creating a WBAN is directly dependent on the data collected by the wearable sensors and its intended medical application [45]. Wearable sensors used in mHealth systems can be classified as follows:

- **Bio-potential sensors**: these sensors are called electrodes and they are used to acquire electrical signals generated by different types of tissue. The surface electrodes consist of a metallic part, usually made from Ag/AgCl, which comes into contact with the skin. Electrodes can be integrated into patches that are applied to the skin or even clothes, creating so-called smart clothes [46]. In mHealth systems, they are used to record ECG, EMG, EEG, and electrooculogram (EOG) signals [47,48,49].
- **Heart-rate sensors**: the most common technology used for implementing these sensors in mHealth systems is photoplethysmography [50,51,52,53]. A light beam (usually with the wavelength of red/infra-red light) is transmitted into the body (e.g., writs, finger, ear lobe, etc.) and usually the intensity of the light is reflected to the receiver component of the sensor. The intensity of the reflected light varies with blood volume variation at the location where the sensor is attached. A PPG signal is obtained that is used to extract the HR. However, a heart-rate can also be extracted from other signals (e.g., ECG) acquired with other types of sensor (e.g., textile electrodes).
- **Blood pressure sensors**: these are used to obtain indirect estimates of systolic pressure (SP), diastolic pressure (DP), mean arterial pressure (MAP), and pulse pressure (PP). Information about blood pressure can be derived from PPG signals [54,55]. Thus, using a sensor for photoplethysmography, heart-rate and blood pressure can be obtained. However, there are sensors for mHealth that are based on the classic oscillometric measurement of blood pressure. A smartwatch can inflate a wrist cuff and, based on the oscillometric method, the SP and DP can be estimated [56,57,58].
- **Respiration rate sensor**: usually, this type of sensor measures strains and is a piezoelectric sensor. The piezoelectric elements are included in a band-aid-like strap that can be put around the thorax, and with every inhalation and exhalation, the thorax moves, deforming the strap, and mechanical stress is applied to piezoelectric elements, generating an electrical voltage. From this electrical signal, the respiration rate can be derived. These sensors can be also included in smart clothes. Other types of sensor measure the difference in air temperature, humidity, or air flow during a breathing cycle (e.g., warmer air during expiration than during inspiration), and the respiration rate is estimated. These sensors can be integrated into face masks [59,60,61,62]. Also, more information about respiration function can be obtained using sensors integrated into smart face masks, such as respiratory minute volume, tidal volume, peak flow rate, and unique respiration patterns [63].
- **Sweat-based wearable electrochemical sensors**: these sensors are used to obtain the following analytes: glucose, alcohol, lactate, pH, vitamin C, $Na^{+}$, and $K^{+}$, and can be integrated in smart glasses, smart bracelets, and smart clothes (e.g., gloves or socks) [64,65,66,67].
- **Galvanic skin response sensors**: these are usually used to estimate the degree of cognitive and emotional arousal based on the electrodermal activity (EDA) or galvanic skin response (GSR). The EDA signal is obtained by placing 2 or 3 electrodes on the skin at hand level (wrist, palm, or fingers). An electrical signal is passed between the electrodes and the resistance is measured. The sweat glands produce more sweat when an emotionally arousing stimulus is experienced, which in turn changes the resistance of the skin. In mHealth applications, GSR sensors can be integrated in smart gloves or smart wristbands [68,69,70,71].
- **Temperature sensors**: these are used to measure human body temperature. Flexible temperature sensors can be easily integrated into mHealth systems and are usually made from temperature-sensitive conductors or inks based on metals, nanomaterials, or conductive polymers [72].
- **Body motion tracking sensors**: these types of sensors are very widespread in mHealth applications for monitoring daily physical activity by tracking step count or by identifying the type of physical exercise performed. They can also be used to monitor body position, identify falls and assess fall risk in older people, or in patients during walking rehabilitation [73,74,75]. Changes in velocity, acceleration, body position patterns, which are used to map body movement, are determined from signals acquired with different types of sensors, including barometers, magnetometers, accelerometers, and gyroscopes. Also, a combination of two or more sensors are used to provide a more precise record of body motion.

### 2.2. Challenges in mHealth Systems

mHealth is a growing domain that has already started to reshape the healthcare system as we know it, and is entirely dependent on advances in wearable sensor, communication, and cybersecurity technology. There are still barriers that must be overcome, including energy consumption, system failure, patient security, data security, data accuracy, data collection infrastructure to manage the high volume of patient data, etc.

Although mHealth approaches are starting to be used in almost all healthcare domains, the evaluation of the clinical impact is still in its early stages [2]. There is evidence to demonstrate that mHealth improves health outcomes, based on 234 clinical trials described in the literature [2].

**Figure 2:**
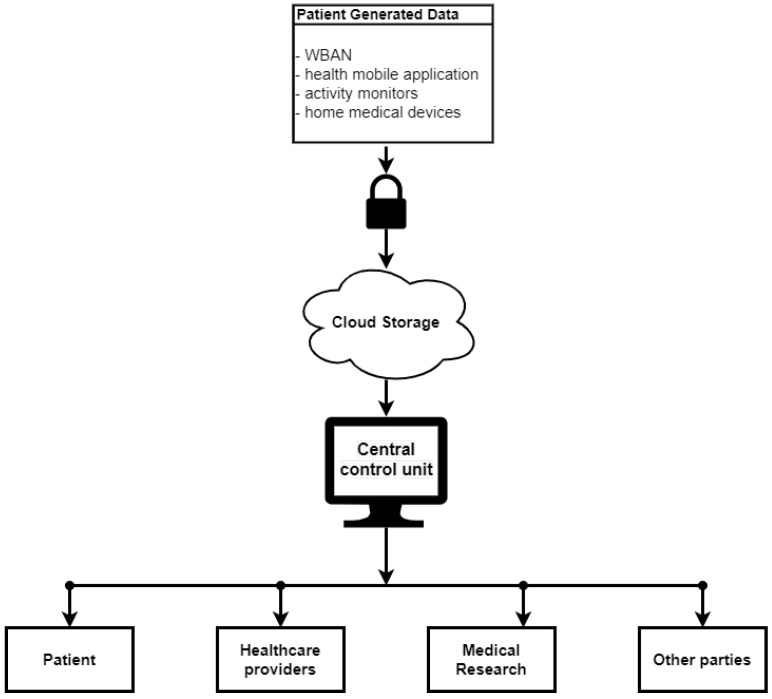
Common mHealth model.

Presently, the traditional model of an mHealth system uses a centralized monitoring unit (Figure 2). This can create critical vulnerabilities for mHealth systems. The foremost challenges related to mHealth systems are system failure, security, and privacy of the patient data. Thus, a vulnerability in security can easily lead to patient health information alteration and theft [76]. Moreover, cybercriminals can achieve complete control of wearable devices and pose a threat to a patient’s life. A case is well known of the insulin pump commercialized by Johnson and Johnson that presented a low security vulnerability permitting hackers to change the insulin dose. For an mHealth system, where wearable devices are interconnected and based on IoT, such a vulnerable device can pose a danger for the entire network, which would lead to the gaining of access to and control of other devices in the network, and ultimately access to the entire health record of the patient. Moreover, it will make the entire mHealth system vulnerable against other types of cybercrimes, such as impersonation, data theft, eavesdropping, etc. [4]. Device-sharing (sharing the device used for mHealth with family and friends) and the lack of cyber-hygiene routines (not using passcodes, encryption, or secure clouds in daily working practice) can also lead to data breaches and unintended consequences [77].

Another important challenge is that wearable devices usually have their own data formats and standards, making the integration of all the data into a mHealth system an important challenge. Also, there is a lack of regulation and guidelines for implementing mHealth approaches [12].

System failures are also highly relevant in the mHealth context, mainly due to the increasing complexity and dynamics of such systems. Some examples of system failures include device failure, overload software failures, and network component failure. These can have impact on patient data availability, real-time collection of patient data, and availability of a system or service, etc. Due to the centralized model of current mHealth systems, all data can become inaccessible, corrupt, or lost.

## 3. Critical Analysis of Blockchain Technology

Blockchain technology was first introduced in 2008, and its best-known use-case is in the cryptocurrency Bitcoin [78] with 516,981 million transactions (as of 1 April 2020) [79]. Apart from this digital currency revolution, other research areas have been influenced by this technology, such as supply chains, the automotive industry, healthcare, smart grids, etc.

**Figure 3:**
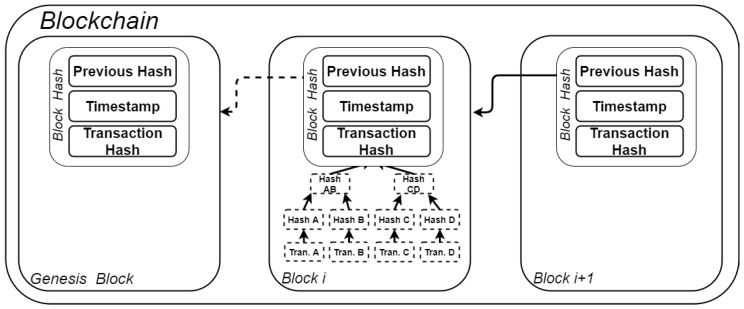
Basic blockchain structure with Merkle tree of transactions for *block i*.

Using blockchain technology, information can be shared between users, and every transaction is recorded and stored locally by each user, thus eliminating the need for a central authority. Because information is logged in a distributed manner, the blockchain can be defined as a fully auditable, digital decentralized ledger of transactions. Each block in the chain can have multiple transactions, and is linked to the previous one by means of cryptographic hash functions (as depicted in Figure 3). The transactions of each block are formed as a Merkle tree, where each leaf value (transaction) can be verified to the known root [80]. Only the root of the tree is recorded in the block. Hence, a new block in the chain will always include a reference to the hash of the previous block. The hash of the new block is computed by the so-called *miners* or *nodes* (participants in the network) and must respect specific rules given by the consensus protocols. Once a valid hash is found, it is broadcast to all participants over the network for validation. After all the nodes have confirmed the validity of the found hash, the new block is added to the chain. At this point, the transaction is immutable.

The miners (the nodes) of the blockchain can have different roles [81]:

- **Lightweight miners**: keep only the header of each block in its local storage;
- **Full miners**: store a complete and current replica of the canonical blockchain locally and autonomously verify the transactions without external reference [81];
- **Consensus miners**: influence the state of the canonical blockchain by publishing new blocks.

Blockchains can be classified into three categories, regarding who is allowed to access, write, and read information:

- **Permisionless or public**: in a public blockchain, anyone can join the network, can write, read, and access all the information in the chain. Moreover, anyone can contribute to the consensus and to the core software [78]. An example of applications that use permisionless blockchains are cryptocurrencies Bitcoin [78] and Ethereum [82].
- **Public permissioned or consortium**: in a consortium blockchain, the identities of all participants are known, and it is open to only limited participants, i.e., only selected groups have the permission to view and can take part in the consensus mechanism. Hence, to validate a new block, it must contain approval from a minimum number of members (the selected groups). A semi-private blockchain or a consortium can be used, for example, by companies that need to interact and share information with other companies and public entities.
- **Permissioned or private**: In a private blockchain, the selected participants, for which the network is open, are usually managed by a central authority.

One of the critical components of the blockchain is the mechanism used to validate and accept new data entries into the distributed ledger. This is usually solved using different consensus protocols such as:

- **Proof of work (PoW)**: when this protocol applies, to add and validate a new data block, the miners compete in finding a hash (i.e., the target block header) of the proposed transaction (block), which is lower than a target value [81]. The first miner that finds the hash that validates the new block is rewarded. However, PoW requires high computational resources, reflected in high economic costs (in particular electricity consumption). It is used and integrated in Bitcoin and many other cryptocurrencies;
- **Proof of Stake (PoS)**: this is a modified version of the PoW protocol that aims to reduce energy draining due to extensive hash queries. When this protocol is applied, each miner has an associated stake, which is measured with so-called *coin age*. A miner basically must solve a PoW puzzle with a specific difficulty, but he can consume his *coin age* to reduce the difficulty of the puzzle solution. However, so as not to give an unfair advantage to the miner with the highest *coin age*, different hybrid PoS versions have been developed, where the stake is combined with some randomization [81];
- **Practical Byzantine Fault Tolerance (PBFT)**: Byzantine Fault Tolerance (BFT) is the characteristic of a distributed network to reach consensus even when some miners in the network respond with incorrect information or fail to respond. Thus, a collective decision is employed with the objective of reducing the influence of faulty miners. The PBFT protocol is applied for permissioned blockchain networks, where a consensus is reached among a small group of known miners. PBFT is currently used in Hyperledger Fabric.

There are also blockchain implementations that support *smart contracts* (e.g., Ethereum). A smart contract is a set of code lines (computer code) that is self-executing, containing a set of pre-specified rules (contractual agreements) that must be satisfied during a blockchain transaction. Hence, because through smart contracts all or parts of pre-agreed rules are executed, the performance of credible transactions without third parties is allowed. To summarize, the main characteristics and benefits of blockchain technology are:

- **Decentralization**: in the blockchain, all transactions are recorded in a shared and decentralized ledger. Hence, a copy of the ledger is present in every node (user) of the blockchain. Moreover, any new transaction is accepted in the chain using various consensus mechanisms; thus, the content of the blockchain is not controlled by a trusted central authority.
- **Immutability and security**: once a transaction is validated on the blockchain, it is almost impossible to change it, mainly due to the decentralized manner in which information is stored. This characteristic offers *data integrity* for the data saved in a blockchain-based system. Thus, if a hacker wants to falsify the data, they will need to make a change in the majority of the blockchain nodes, which is almost impossible, so the blockchain has an inherent high degree of *security*.
- **Transparency and privacy**: it can offer different degrees of transparency, from public blockchains, where any user is allowed to join the network and anyone can see the data stored, to more private blockchains where a user needs permission to create transactions with other users, and all users are authenticated and known. Privacy is assured by powerful cryptography, making it very difficult to identify a user.
- **Traceability and accountability**: because the technology is based on a chain of blocks where each block is linked to the previous one by including the hash of the latter [80], it offers the possibility to everyone or to an authorized entity to audit the history of all transactions.

### Blockchain Applications in Healthcare

In the specialist literature, blockchain is used in healthcare processes/systems to improve access control, interoperability, data integrity, and data provenance.

According to a recent literature review [80], blockchain technology is used in healthcare mainly for improving processes such as: (a) health data recording, storing, and sharing [83]; (b) remote collection and storage of health data [84]; (c) sharing of healthcare information for clinical, and/or research, and/or administrative (economic) purposes [85,86,87,88,89,90]; (d) managing access to personal health data and EHR [91]; (e) collecting and sharing health-related sensor data for clinical purposes [92]; (f) collection and storage of data for automated diagnostics [93]; (g) automatic collection, storage, and patient-controlled sharing of personal health [94]; (h) sharing healthcare data between health institutions; (i) patient data management and storage in a cloud environment [95]; (j) the recruitment of patients to clinical trials [95]; (k) establishing a patient-controlled marketplace for the selling and buying of healthcare information for research purposes; (l) monitoring the outbreak of infectious diseases; (m) retrieving information in the EHR [96]; (n) patient-controlled collection and sharing of sensor data [97]; (o) decision-making by presenting knowledge [98]; (p) exchange of medical images [99]; (q) finding a patient in the context of telemedicine services [100].

Some of the most popular existing blockchain platforms used in medical applications are Ethereum, Hyperledger Fabric, and Exonum, while the most used blockchain type is public permissioned (consortium), followed by permissioned (private) and public [80]. It seems that a consensus mechanism based on smart contracts is preferred when using blockchain technology in healthcare [80,85,93,101,102].

As reported in [80], the most impacted healthcare information system by blockchain technology is EHR (17 out of 39 papers analyzed), followed by personal health records (6 out of 39 papers analyzed). In just 1 out of 39 papers analyzed, there are healthcare information systems such as picture archiving and communication systems, an automated diagnostic service for patients, population health management system, knowledge infrastructures, pharma supply chain, and IoT data management/personal health data.

Another healthcare domain that is starting to benefit from blockchain technology is mHealth, which also includes patient-controlled collection, and storage and sharing of sensor data [84,94,102,103,104]. With advances in biomedical sensor technology and telemonitoring, and with the need for the remote monitoring of patient health due to safety concerns in recent years (e.g., during the COVID-19 pandemic) and economic reasons, the mHealth field has developed rapidly. However, regulations to health data manipulation resulting from mHealth applications are not yet defined. Blockchain technology is a promising solution for solving different aspects related to data manipulation in the mHealth field such as immutability, security, and privacy.

## 4. Secured and Distributed Mhealth System—Proposed Framework.

### 4.1. Data Description

To test the proposed framework, a collection of multi-parameter physiological signals recorded with wearable sensors is used. The wearable system described in [105] consisted of sensors placed on the body of car drivers to record the physiological signals ECG, HR, EMG, respiration, and foot and hand GSR while they were driving on a predetermined route [105]. Table 1 presents the acquisition parameters for the physiological signals recorded with the wearable sensors.

For hand and foot GSR, two pairs of electrodes are placed on the palm of the hand and the sole of the foot and a small electrical current is passed across the two electrodes to record changes in skin resistance. To record the ECG signal, three electrodes are used to obtain a modified lead II. The respiration signal is derived from measuring thoracic movement due to breathing. For this, a Hall effect sensor is used, which consists of two magnets embedded inside an elastic tube. The EMG signal is acquired using three electrodes, two of which are placed on the axis of the trapezius muscle and the third one is used as a reference [105].

Sixteen data sets are used to test the proposed framework. Each of the data sets has a duration that varies between 65 and 93 min, and contains the 6 physiological signals previously described. In Table 2 the total number of samples and the size of each dataset is presented.

### 4.2. Proposed Framework

#### 4.2.1. Proposed Framework Overview

The goal of the proposed framework is to create a distributed, immutable, and secure environment for patient–doctor interaction through bidirectional medical data exchange. In this section, a proof-of-concept implementation is presented, which allows patients and healthcare professionals to register and share medical information from various data sources, such as wearable devices or professional medical equipment. The main features of the proposed mHealth framework are:

- **Patient/doctor identity**: patients and doctors must register on the framework and their identity and privacy is managed by a blockchain smart contract.
- **Patient/doctor association**: each patient can be attended to by multiple doctors (of various specialties). Patient management is ensured by a blockchain smart contract, and patients retain control over their medical data.
- **Data immutability/integrity**: both patients and doctors can add medical data from various sources, such as wearable devices. The framework is designed to be data-agnostic, allowing for a wide variety of data sources. Data immutability and integrity is ensured by hashing and by the properties of the blockchain network.
- **Data interaction and accountability**: healthcare providers associated with patients can interact with the data through visualization, annotation, and diagnosis. All authorized participants can trace and audit the data origin and history.

**Figure 4:**
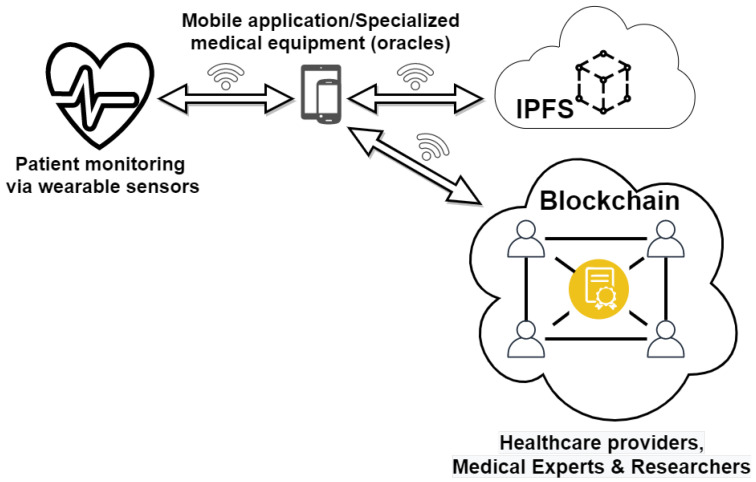
Proposed framework architecture.

The proposed framework is presented in Figure 4. It consists of a decentralized mHealth application that monitors patient parameters via wearable sensors. For this purpose, a private Ethereum blockchain is implemented and deployed, to avoid costs associated with transactions on the public blockchain, as well as to ensure user privacy.

**Figure 5:**
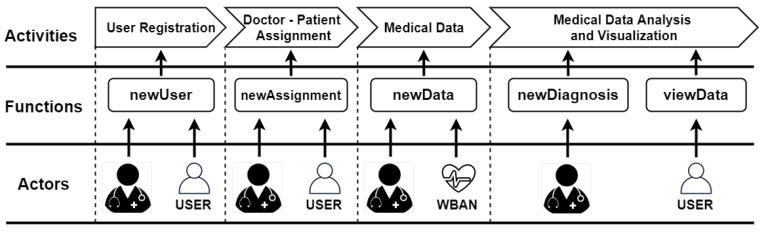
Smart-contract architecture.

Patient information is transmitted directly to the blockchain network and received by a smart contract that implements the necessary functions. In the diagram presented in Figure 4, the healthcare providers, medical experts, and researchers, who should be able to access patient records, act as active (mining) nodes. Patients can connect directly to the blockchain network without a central server or database, thus eliminating single points of failure. Since the amount of raw data that is collected is very high (Table 2), it is inefficient to store all the measurements on the blockchain network. Instead, in the proposed framework, raw data is stored as JSON files on the IPFS, which is a decentralized hypermedia protocol for file storage and remote access. On IPFS, each file receives a unique hash, and the file content is distributed across the network. The file hash is stored on the blockchain and associated with each patient. The structure of the raw JSON dataset is presented below: 
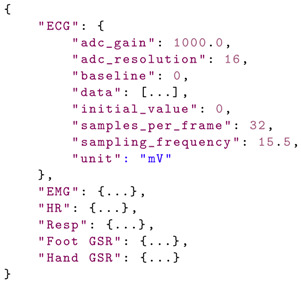


The functionality of the proposed mHealth application is ensured by a smart contract designed, implemented, and deployed on the private Ethereum blockchain network. The architecture of the contract is presented in Figure 5.

#### 4.2.2. Data Model

Patient and doctor information is stored in two separate structures defined in the smart contract and presented below: 
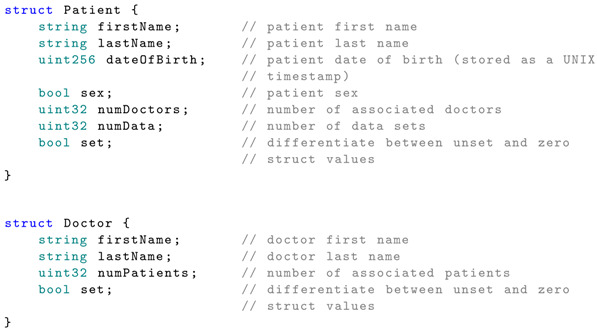


In a traditional database design, there would be a many-to-many (M:M) association between doctors and patients. Doctors or healthcare providers have access only to the records of their associated patients. In the proposed smart contract, the associations between the Ethereum address and the users, as well as the association between doctors and patients, are implemented using mapping. 
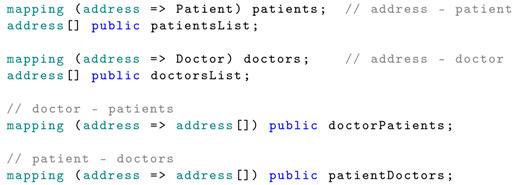


It can be seen that the Patient and Doctor structures contain the properties numDoctors and numPatients. These properties are necessary to iterate through the list of patients associated with a doctor or vice versa, since the Solidity mapping is not iterable.

The data structure and mapping are presented below: 
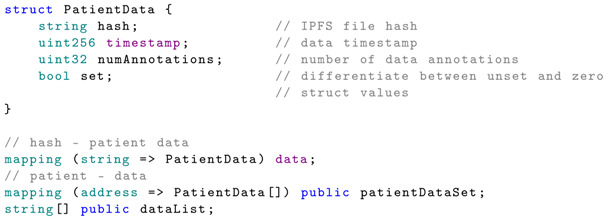


The hash property represents the IPFS file hash. To obtain this, the proposed mHealth application sends the raw JSON data to the IPFS. Once the data is stored, the file hash is returned, which is then stored in the smart contract. The data sets are uniquely identified by their hash. The timestamp of the data measurements is also stored, and the data is associated with the patient using the patientDataSet mapping.

#### 4.2.3. Proposed Framework Implementation

For a patient or healthcare provider to use the proposed mHealth application, they will register on the network (newUser function), according to the user type (patient or doctor). After registration, each user will have a blockchain address generated and associated with their account. The address acts as their identification on the network, as well as their wallet, which could be used for medical service payments in the future. All interactions between users and the proposed framework are done through a web interface that interacts with the private Ethereum blockchain and the IPFS distributed storage protocol through the Web3.py and ipfshttpclient Python libraries. The newUser function emits an event upon completion that will be described further in this section (Algorithm 1).
**Algorithm 1** New user registrationInput: Patient/Doctor personal information: first name, last name, date of birth, sex, user typeOutput: Patient/Doctor address**Require:** User address not already registered1: Generate a new address/private key using the user defined password2: **if** new address does not exist **then**3:     **if** user is patient **then**4:         Store patient information5:     **else**6:         Store doctor information7:     Emit new user event8: **return** address

After the users are registered, the patients and doctors must be associated. A patient may have multiple physicians and a doctor can attend to multiple patients. These associations are created through the newAssignment function (Algorithm 2).
**Algorithm 2** New assignment functionInput: Patient address, doctor address**Require:** Patient and doctor are registered1: Update the patient-doctor mappings with the new association2: Emit new assignment event

Once patients/doctors are registered, they can transmit data from various sources, using a mobile or web application that interacts directly with the proposed framework. In this context, the application will act as an oracle (a trusted source of information) for the blockchain network. New data is added using the newData function (Algorithm 3). Since the data is loosely represented as JSON-encoded strings, various data sources can be easily integrated without modifying the blockchain smart-contract logic (only front-end changes may be required to support new devices or data sources).

**Figure 6:**
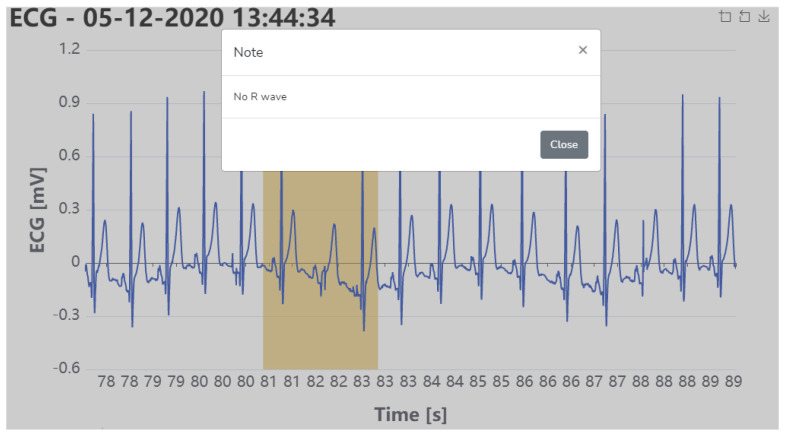
Example of annotations that can be made by the healthcare providers.

The physician can add annotations, diagnoses, or treatment recommendations to the data (newDiagnosis function) (Figure 6), which can be received and viewed by the patient or other healthcare providers (viewData function), according to Figure 5 (Algorithm 4).

The events emitted by each smart-contract-writing function are an integral part of distributed application development. They allow communication between smart contracts and user interfaces, acting as data triggers, so they can be considered to be an API for the smart contract. The associated applications can subscribe to one or more smart-contract events, and the necessary logic can be implemented (e.g., patient/doctor notification, problem highlighting, etc.). Although it is not required that the smart-contract functions emit these events, they greatly improve the process of blockchain application development.
**Algorithm 3** New data functionInput: Patient address, IPFS file hash, timestamp**Require:** Patient is registered**Require:** Data file is not already saved1: Store JSON file to IPFS and retrieve file hash2: Store file hash and timestamp on the blockchain3: Update the patient - data mapping4: Emit new data event5: **return** IPFS file hash
**Algorithm 4** New diagnosis functionInput: Patient address, doctor address, dataset hash, diagnosis file hash, timestamp**Require:** Patient and doctor are registered**Require:** Doctor is associated with the patient**Require:** The patient dataset hash exists1: Store diagnosis file to IPFS and retrieve file hash2: Store diagnosis file hash and timestamp on the blockchain3: Update the dataset—diagnosis and doctor—diagnosis mappings4: Emit new diagnosis event5: **return** Diagnosis file hash

The proposed framework was deployed on three nodes with the following configurations (Table 3):

In the proposed implementation, all three nodes are configured as mining nodes on the private Ethereum blockchain. In the context of the proposed framework, it is not necessary for the patients to run the blockchain nodes and sync with the blockchain network. Only physicians and healthcare providers must synchronize with the blockchain ledger and reach consensus, while the patients interact with the mHealth network through desktop or mobile applications which transmit information to the blockchain smart contract and interact with the data through events and logs. For the proposed proof-of-concept implementation, a Python web application is developed, but this can be bundled as a standalone application. It is important to note that this application does not require any server-side components (a centralized server is not required), so the proposed mHealth framework is a fully decentralized application.

**Figure 7:**
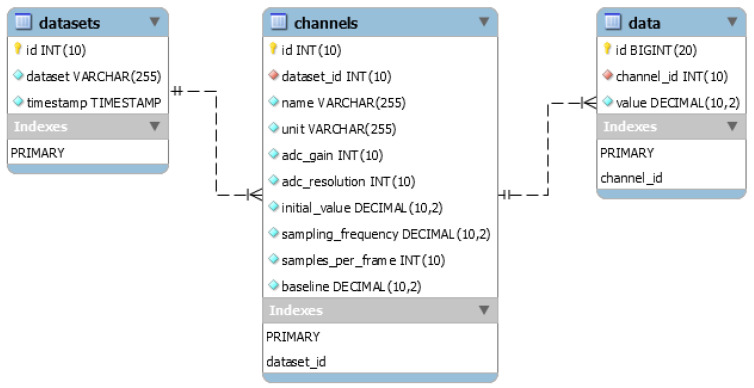
MySQL database used for performance evaluation.

To benchmark the proposed mHealth application performance, the saving times for the datasets in Table 2 are evaluated and compared to a classical centralized database approach, using a relational MySQL database (Figure 7).

## 5. Results

**Figure 8:**
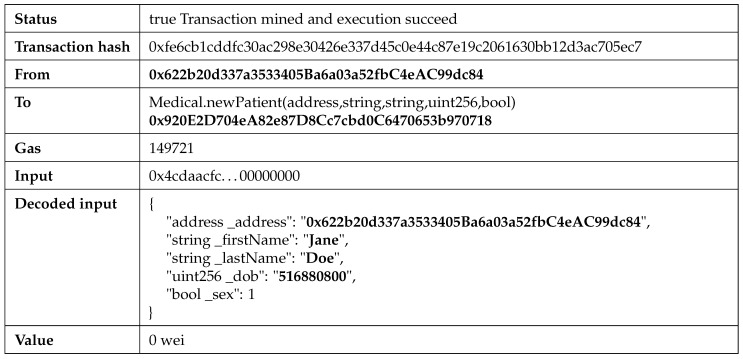
New patient transaction on the Ethereum blockchain.

Each operation depicted in Figure 5 represents a transaction on the Ethereum blockchain. Once a transaction is mined and confirmed on the network, it will be included in a new block, as described in Section 3. The structure of a new patient registration transaction is shown in Figure 8.

The registration transaction executes the newPatient smart-contract function. The Input represents the byte-encoded function name and parameter values for the function call, which is decoded in the Decoded input field. The gas required for this transaction is 149,721, which corresponds to 0.018565404 ETH (considering the gas price of 1 gas unit = 124 GWei). If the smart-contract function were executed on the public Ethereum blockchain, the transaction fee would have resulted in the equivalent of 33.60 USD, considering the Ethereum value of 1810 USD (as of 13.02.2021). Using a private blockchain, the token values and transaction fees can be either ignored or they can be used to implement an in-app payment system in the future, where the token and fee values can be fixed and not be influenced by existing cryptocurrency fluctuations.

**Figure 9:**
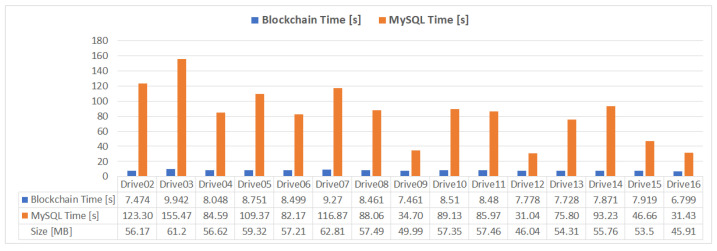
Blockchain and MySQL write times.

**Figure 10:**
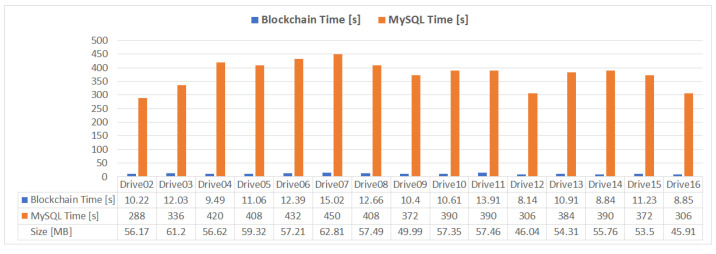
Blockchain and MySQL read times.

The write and read results of the data using the proposed implementation and the MySQL database (Figure 7) are presented in Figure 9 and Figure 10. It can be observed that the write time required for a relational database approach, using optimized batch INSERT queries for data insertion, is between 4 and 16 times higher than the times obtained with the proposed IPFS JSON storage solution, while the read times necessary to create the same JSON structure presented in Section 4.2 are between 28 and 45 times slower than the times obtained with the proposed solution. For both tests, all the samples in each dataset presented in Table 2 were included in the measurement.

**Figure 11:**
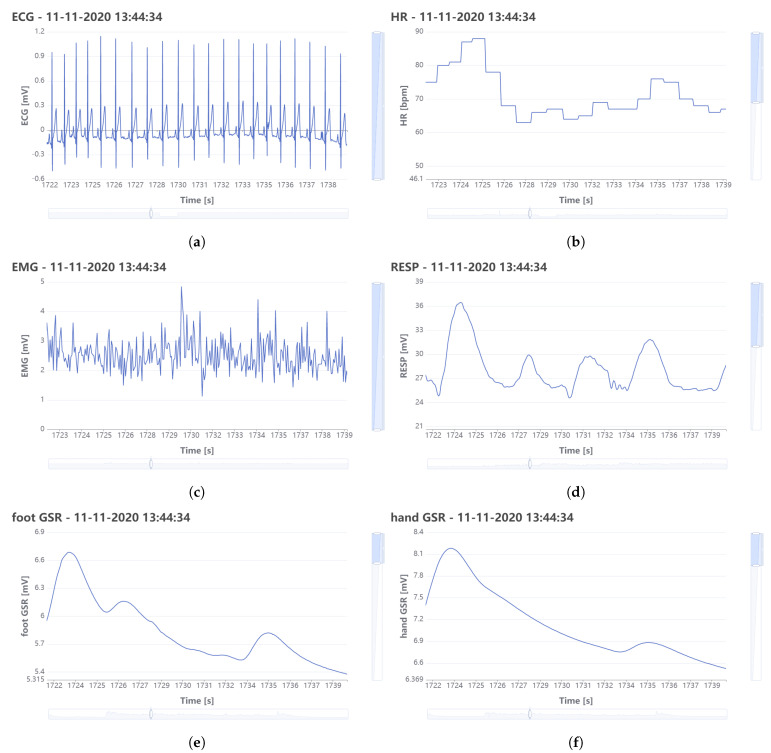
Physiological signals recorded with wearable sensors: (**a**) ECG; (**b**) HR; (**c**) EMG; (**d**) Respiration; (**e**) foot GSR; (**f**) hand GSR.

An example of the physiological signals recorded for one driver is depicted in Figure 11 and the signals are represented as they appear in the web interface created for the proposed framework, where the physicians can interact with the signals by means of measurements and annotations.

## 6. Discussion

Table 4 compares the proposed blockchain framework with the existing ones described in the literature. Griggs et al. [102] introduced a system based on a private Ethereum blockchain with smart contracts. A sensor, such as a heart-rate monitor, records and sends data to a smart device, such as smartphone or tablet, which in turns send the data to the smart contract. The smart contract is executed and evaluates the data and in turn can send alerts to the patient, hospital, or medical expert, or can send a command to activate medical devices such as insulin pumps. In [103], the authors proposed a decentralized privacy-preserving healthcare blockchain for IoT. The logical flow execution is similar to the one introduced in [102]. It uses symmetric and asymmetric encryption schemes and lightweight digital signatures for authentication purposes [103].

Zhang et al. imagined a model for sharing medical data through social network nodes. In this model, the data is generated by a WBAN. Using an improved version of the IEEE 802.15.6 standard, the nodes in the network can set up secure connections using sensor and mobile device addresses that are stored on the blockchain. All the data is stored in the smart devices and body sensors [84].

In [95] Wang et al. proposes an Ethereum-based framework with attribute-based encryption (ABE) technology to obtain access control over EHR data that is stored in the decentralized system. No private key generator (PKG) is used and the encryption key of the file with medical data is stored on the blockchain using the Advanced Encryption (AES) algorithm [95].

Some of the main limitations of the frameworks discussed include (a) no experiments or simulations, just conceptual analysis; (b) storage of the medical data directly in the blockchain which is not feasible for a complete mHealth system with a WBAN, or the medical data is stored in a centralized database or directly on the smart devices/sensors, which makes the medical data vulnerable to data loss, alteration, and theft; (c) no interface implemented for the medical experts, not only to visualize medical data but also to directly interact with it to obtain diagnostic information. Although there are algorithms for the deployment of blockchain that can improve healthcare processes, there is a need to improve medical diagnosis, i.e., to use the conceptual knowledge of blockchain applications for specific issues, such as diagnostics [106].

The proposed framework overcomes these limitations. The medical data that comes from the WBAN is not stored directly on the blockchain, but instead is stored in a decentralized data-storage system, assuring protection to data alteration, theft, or loss. Only the hash key for each dataset is stored on the blockchain. Using the smart contract, the association between the patient, the dataset, and the medical expert is realized. Moreover, the proposed framework is not a conceptual analysis; instead, the blockchain and smart contracts are implemented, deployed, and tested using real signals collected by wearable sensors. In addition, the framework also offers an interface for both patient and medical experts. The latter provides capabilities for direct interaction with the medical data. Thus, the medical expert can visualize and manipulate the signals recorded by sensors to make a precise diagnosis. It has also the possibility to transmit alerts, treatment and therapy recommendations to the patient. All are stored in the electronic record of the patient.

Another advantage of the proposed approach is that blockchain network nodes are represented only by healthcare professionals, thus effectively replacing a centralized EHR database with a distributed ledger. The first benefit of this approach is that patients do not need to synchronize the blockchain ledger and have a full node connected to the blockchain network. Due to the way the smart contract is developed, authorized applications (oracles) can be integrated into the mHealth environment. The second benefit of the proposed approach is that the consensus mechanism can be changed from the current proof-of-work method to a proof-of-stake or proof-of-authority consensus mechanism, since only medical professionals actively participate in data validation and consensus. This would require fewer resources than the current proof-of-work consensus implemented in the Ethereum blockchain. A detailed analysis of the performance impact of the three consensus mechanisms will be addressed in a future study, since Ethereum already supports proof-of-authority consensus (on the Rinkeby test network and on private networks using the Clique consensus protocol) and will soon support the proof-of-stake consensus as well.

It is well known that blockchain smart-contract changes require a lot of operations under the hood, since as with every other transaction on the blockchain, the smart contract is immutable once it is deployed. Any change to the smart-contract logic implies the redeployment of a new contract instance. However, the data must be referenced or transferred from the previous instance. Due to the data-agnostic approach of the proposed framework, no contract changes are necessary when the data format is modified. Using IPFS as a decentralized solution for the storage and hashing of the data by content achieves two important performance improvements: (a) data duplication is avoided, since the same content will produce the same hash on the IPFS network, thus eliminating unwanted redundancy and optimizing the amount of transferred data; and (b) the data format is not coupled with the blockchain smart-contract logic, so new data sources and formats can easily be added, by updating the front-end application to support the new JSON format. Updating a client-side application is much more convenient, through various distribution channels available for desktop, mobile, or web applications.

## 7. Conclusions

In this paper, an mHealth application for remote patient monitoring is presented using blockchain technology to create a decentralized and distributed network of healthcare providers and patients that can share and interpret medical data. Using a privately deployed Ethereum blockchain, both patients and healthcare providers can join the mHealth network, through a web interface, which allows the sharing, viewing, and interpretation of medical records. A smart contract deployed on the Ethereum blockchain is responsible for data management and proper association between doctors, patients, and monitored data. Since blockchain operations are expensive in terms of storage and processing, the medical data is distributively stored using the IPFS protocol, creating additional redundancy and data availability. Traditionally, a remote, web-based monitoring system would be implemented using a client–server model, where the data would be stored in a centralized database (most likely a SQL variant, such as MySQL). This centralized approach is susceptible to data loss due to server failure or third-party attack. Due to the large number of collected samples, the centralized database approach is much slower than the proposed solution in this paper, as shown in Figure 9 and Figure 10. Although some optimization could be made to the data model and the querying techniques, a database-driven approach would still be susceptible to a single point of failure. Using blockchain, the information is distributed across all nodes in the network, thus improving the data availability and transfer times.

A future version of the proposed framework could also allow payment options for medical services or for accessing patient health data (e.g., for clinical studies, etc.). Since most blockchain platforms allow the transfer of value, in the form of network tokens, a privately deployed blockchain network can use this mechanism to implement payment options, without requiring additional components, while the JSON storage approach can allow for different devices (professional or recreational wearable devices) to be used and integrated with ease.

## Figures and Tables

**Table 1:** Acquisition parameters for the physiological signals.

Signals	Gain	Resolution	Samples/Frame	Sampling Frequency
ECG	4000	16	32	15.5
EMG	4000	16	1	15.5
HR	1	16	1	15.5
Resp	100	16	2	15.5
Foot GSR	1000	16	2	15.5
Hand GSR	1000	16	2	15.5

**Table 2:** Data sets.

Dataset	Number of Samples	Size (MB)	Dataset	Number of Samples	Size (MB)
Drive01	10,208,834	195.74	Drive09	2,702,392	49.98
Drive02	2,966,128	56.17	Drive10	3,099,272	57.34
Drive03	3,276,493	61.2	Drive11	3,096,853	57.46
Drive04	3,050,640	56.61	Drive12	2,472,224	46.03
Drive05	3,213,047	59.32	Drive13	2,918,604	54.30
Drive06	3,080,043	57.20	Drive14	2,993,440	55.76
Drive07	3,380,122	62.8	Drive15	2,888,360	53.5
Drive08	3,095,869	57.48	Drive16	2,477,138	45.9

**Table 3:** Proposed framework node configurations

Component	Node 1	Node 2	Node 3
CPU	Quad core AMD Athlon X4 860K @ 3.7 GHz	Quad core Intel i5-4400 @ 3.1 GHz	Triple core AMD Phenom X3 8750 @ 2.4 GHz
Memory	16 GB	4 GB	4 GB
Operating system	Ubuntu Linux 18.04 LTS	Ubuntu Linux 18.04 LTS	Ubuntu Linux 18.04 LTS
Geth version	1.9.25	1.9.25	1.9.25
Python version	3.9.2	3.6.9	3.6.9

**Table 4:** A comparative analysis of the proposed framework vs the state-of-the-art systems

Blockchain Platform for mHealth System	Patient Identity	Immutability	Data Audit	Authentication	Accountability	Data Integrity	Interactive Data Evaluation
Proposed framework	√	√	√	√	√	√	√
Griggs et al. [102]	√	partial	√	√	√	partial	
Dwivedi et al. [103]	√	√	√	√	√	√	
Zhang et al. [84]	√	√	√	√	√	√	
Wang et al. [95]	√	√		√		√	
